# Effects of Ambient Coarse, Fine, and Ultrafine Particles and Their Biological Constituents on Systemic Biomarkers: A Controlled Human Exposure Study

**DOI:** 10.1289/ehp.1408387

**Published:** 2015-01-16

**Authors:** Ling Liu, Bruce Urch, Raymond Poon, Mieczyslaw Szyszkowicz, Mary Speck, Diane R. Gold, Amanda J. Wheeler, James A. Scott, Jeffrey R. Brook, Peter S. Thorne, Frances S. Silverman

**Affiliations:** 1 Healthy Environments and Consumer Safety Branch, Health Canada, Ottawa, Ontario, Canada; 2 Gage Occupational and Environmental Health Unit, Department of Medicine, Faculty of Medicine, University of Toronto, Toronto, Ontario, Canada; 3 Southern Ontario Centre for Atmospheric Aerosol Research (SOCAAR), Toronto, Ontario, Canada; 4 Li Ka Shing Knowledge Institute, St. Michael’s Hospital, Toronto, Ontario, Canada; 5 Channing Laboratory, Brigham and Women’s Hospital, Harvard Medical School, Boston, Massachusetts, USA; 6 Centre for Ecosystem Management, School of Natural Science, Edith Cowan University, Joondalup, Western Australia, Australia; 7 Dalla Lana School of Public Health, University of Toronto, Toronto, Ontario, Canada; 8 Environment Canada, Toronto, Ontario, Canada; 9 Department of Occupational and Environmental Health, University of Iowa, Iowa City, Iowa, USA

## Abstract

**Background:**

Ambient coarse, fine, and ultrafine particles have been associated with mortality and morbidity. Few studies have compared how various particle size fractions affect systemic biomarkers.

**Objectives:**

We examined changes of blood and urinary biomarkers following exposures to three particle sizes.

**Methods:**

Fifty healthy nonsmoking volunteers, mean age of 28 years, were exposed to coarse (2.5–10 μm; mean, 213 μg/m^3^) and fine (0.15–2.5 μm; mean, 238 μg/m^3^) concentrated ambient particles (CAPs), and filtered ambient and/or medical air. Twenty-five participants were exposed to ultrafine CAP (< 0.3 μm; mean, 136 μg/m^3^) and filtered medical air. Exposures lasted 130 min, separated by ≥ 2 weeks. Blood/urine samples were collected preexposure and 1 hr and 21 hr postexposure to determine blood interleukin-6 and C-reactive protein (inflammation), endothelin-1 and vascular endothelial growth factor (VEGF; vascular mediators), and malondialdehyde (lipid peroxidation); as well as urinary VEGF, 8-hydroxy-deoxy-guanosine (DNA oxidation), and malondialdehyde. Mixed-model regressions assessed pre- and postexposure differences.

**Results:**

One hour postexposure, for every 100-μg/m^3^ increase, coarse CAP was associated with increased blood VEGF (2.41 pg/mL; 95% CI: 0.41, 4.40) in models adjusted for O_3,_ fine CAP with increased urinary malondialdehyde in single- (0.31 nmol/mg creatinine; 95% CI: 0.02, 0.60) and two-pollutant models, and ultrafine CAP with increased urinary 8-hydroxydeoxyguanosine in single- (0.69 ng/mg creatinine; 95% CI: 0.09, 1.29) and two-pollutant models, lasting < 21 hr. Endotoxin was significantly associated with biomarker changes similar to those found with CAPs.

**Conclusions:**

Ambient particles with various sizes/constituents may influence systemic biomarkers differently. Endotoxin in ambient particles may contribute to vascular mediator changes and oxidative stress.

**Citation:**

Liu L, Urch B, Poon R, Szyszkowicz M, Speck M, Gold DR, Wheeler AJ, Scott JA, Brook JR, Thorne PS, Silverman FS. 2015. Effects of ambient coarse, fine, and ultrafine particles and their biological constituents on systemic biomarkers: a controlled human exposure study. Environ Health Perspect 123:534–540; http://dx.doi.org/10.1289/ehp.1408387

## Introduction

Urban particulate matter (PM) in ambient air is a complex mixture of various sizes of particles, and is generally categorized into coarse [mass median aerodynamic diameter (MMAD) 2.5–10 μm; PM_10–2.5_], fine (MMAD ≤ 2.5 μm; PM_2.5_), and ultrafine (MMAD ≤ 0.1 μm) particles. In urban centers, sources of PM_2.5_ and ultrafine particles typically originate from mobile and industrial fossil fuel combustion, whereas PM_10–2.5_ often originates from dust and soil as well as mechanical abrasive processes from industrial and transportation sectors (HEI Review Panel on Ultrafine Particles 2013). Coarse PM often contains bacterial constituents (Schins et al. 2004). The adverse health effects of PM_2.5_ in ambient air on population mortality and hospital admissions have been extensively studied and reviewed (World Health Organization 2013). Many changes in lung and cardiovascular physiology (Liu et al. 2009a; Pope et al. 2004) and biological mediators in blood (Chuang et al. 2007; Delfino et al. 2009; Rückerl et al. 2007) have also been observed on high PM_2.5_ concentration days in epidemiological studies.

Recent studies have reported the mortality and morbidity effects of PM_10–2.5_ (Stafoggia et al. 2013; Zanobetti and Schwartz 2009) and ultrafine particles (HEI Review Panel on Ultrafine Particles 2013). Relative to fine PM, few studies have documented the associations between exposure to PM_10–2.5_ or ultrafine particles and airway inflammation and systemic outcomes in epidemiological studies (Lipsett et al. 2006; Pekkanen et al. 2002) or controlled human exposure studies (Behbod et al. 2013; Brook et al. 2013; Graff et al. 2009; Samet et al. 2009). Few studies have compared how these ambient particle size fractions affect systemic inflammation and oxidative stress in the human body. A toxicological study has shown that on a comparative mass basis, coarse, fine, and ultrafine PM affected murine lungs and hearts in a different manner, with coarse PM more potently influencing airway inflammation and ultrafine particles more potently influencing cardiac function (Cho et al. 2009). Samet et al. (2007) summarized findings from their controlled human exposure studies: Exposures to concentrated ambient PM_2.5_ and PM_10–2.5_ were associated with increased airway inflammation and a trend of increased blood coagulation markers such as fibrinogen and plasminogen, but exposure to ultrafine particles had no such effects.

Endotoxin is a major constituent of the outer membrane of the cell wall of gram-negative bacteria, and in blood triggers the signaling cascade for macrophage/endothelial cells to secrete proinflammatory cytokines (Copeland et al. 2005). β-1,3-d-glucan (glucan) comes from the cell wall of fungi and plants, and is known to be a modulator of immune system function (Fogelmark et al. 1994). Although endotoxin and glucan in indoor dust have been associated with respiratory illness in children (Dales et al. 2006; Douwes et al. 2000), little is known about the adverse health effects of these biological constituents in outdoor PM.

In this study, we hypothesized that *a*) a short-term exposure to concentrated ambient coarse, fine, or ultrafine PM would be associated with increased systemic inflammation, oxidative stress, and changes in mediators of vascular function in healthy individuals, detectable through biomarkers; and *b*) primary biological material in these particles might play a role in concentrated ambient particle (CAP)–related systemic effects.

## Materials and Methods

*Study participants*. The study design was a single-blind randomized crossover trial. We recruited participants through advertising on a local university campus. Participants were nonsmokers, 18–60 years of age, without history of coronary artery disease, myocardial infarction, peripheral vascular disease, angina, heart failure, hypertension, or diabetes mellitus. All participants were free of lipid abnormalities and respiratory tract infections. We excluded participants with baseline spirometry < 75% of predicted normal values (forced vital capacity and forced expiratory 1-sec volume), having clinically significant abnormalities in their resting electrocardiogram, or being pregnant or breastfeeding. All participants provided informed written consent prior to participating in the study. The Research Ethics Boards of Health Canada, St. Michael’s Hospital, and the University of Toronto approved the study protocol.

*Exposure facility*. Details of the coarse, fine, and ultrafine particle concentrator facility were described elsewhere (Rastogi et al. 2012). The controlled exposures to CAPs drew air from breathing height adjacent to a downtown street in Toronto, Canada. We used the Harvard Ambient Fine and Coarse Particle Concentrators (Harvard School of Public Health) and an enclosed temperature-controlled exposure chamber for the study participants. Ambient aerosols were drawn through a size-selective inlet where particles >10 μm were removed. The fine PM concentrator delivered CAP 0.15–2.5 μm in MMAD (fine CAP), and the coarse PM concentrator delivered CAP 2.5–10 μm in MMAD (coarse CAP). Particle-free filtered ambient air (FA) was used as a control by inserting a high-efficiency particulate absorption (HEPA) filter inline downstream of the particle concentrator. For the study on coarse and fine CAPs, we enrolled 50 participants. Early in the study, we observed a pattern of similar postexposure changes in outcome measures (for example, blood pressure and blood neutrophils) for FA and CAP exposures. We hypothesized that ambient gaseous pollutants may have contributed to the FA responses. To explore this possibility, for another exposure to 41 participants, we added HEPA-filtered cylinder medical air that was particle- and ambient gas–free. The study for the coarse and fine CAPs included up to five exposures for each participant when he or she was available: two exposures to coarse CAP, and one exposure to fine CAP, HEPA-filtered ambient air, and/or filtered medical air.

In the airstream of the ultrafine particle concentrator, particles > 0.3 μm were removed by inertial impaction to deliver a concentrated ultrafine aerosol to the participant. For the study on ultrafine CAP, we used the HEPA-filtered medical air as control. Twenty-five participants completed the ultrafine CAP study, 20 of whom also participated in the coarse and fine CAP study.

The exposure air stream was delivered directly to the participant who was seated at rest and breathing freely via an “oxygen type” face mask covering his/her nose and mouth. Each exposure lasted 130 min. PM in the airstream was collected on a filter during the 130-min exposure, and gravimetric determinations of mass concentrations reported. Between exposures there was a washout period of ≥ 2 weeks.

*Gaseous pollutant monitoring*. We measured concentrations of gaseous pollutants in the exposure facility in the airstream delivered to the participant. Gases measured included sulfur dioxide (SO_2_, fluorescent; Monitor Labs model 8850), ozone (O_3_, ultraviolet photometric; Dasibi model 1008RS), nitrogen dioxide (NO_2_, chemiluminescent; Monitor Labs model 8840), and carbon monoxide (CO, infrared; Thermo Electron Instruments model 49). We reported the mean of the 15-sec averages for the 130-min exposure period.

*Measurement of endotoxin and glucan*. Detailed methods for the measurements of endotoxin and glucan were previously described (Behbod et al. 2013). Endotoxin and glucan were collected on polycarbonate membrane filters during exposures to CAPs and filtered ambient air (but not filtered medical air). Natural log transformation was performed on endotoxin and glucan concentrations [ln(value+1)] to obtain a normal distribution of the data.

*Measurement of biomarkers in blood and urine*. We collected urine and venous blood samples (20 mL) before and at 1 hr and 21 hr after each exposure. Upon participants’ arrival for the first exposure, their preexposure blood and urine samples were used to determine baseline values. We measured their height and weight and calculated body mass index (BMI) using the standard procedures.

Blood tests. We obtained fasting blood samples by venipuncture and stored plasma at –70°C. High sensitivity C-reactive protein (CRP) was analyzed using ELISA kit from Alpco Laboratory Products Company (Salem, NH, USA); interleukin-6 (IL-6), endothelin-1 (ET-1), and vascular endothelial growth factor (VEGF) were analyzed using ELISA kits from R&D Systems (Minneapolis, MN, USA). Malondialdehyde (MDA) was measured using high-performance liquid chromatography (HPLC) with an Agilent 1200 series system (Agilent Technologies, Mississauga, Ontario, Canada).

Urine tests. We collected and stored urine samples at –20°C. Urine samples were clarified by centrifugation (5,000 rpm, 5 min in an Eppendorf 5804 centrifuge) before analyses. VEGF and 8-hydroxydeoxyguanosine (8-OHdG) were measured using ELISA kits (8-OHdG kit; Cosmo Bio USA, Carlsbad, CA, USA). HPLC analysis was used to measure urinary MDA. Creatinine concentrations were measured using a CREA kit (Roche Diagnostics, Laval, Quebec, Canada) to normalize urinary biomarker concentrations.

*Statistical analysis*. We tested statistically significant differences in concentrations of endotoxin, glucan, and gases among exposure scenarios using Kruskal–Wallis one-way analysis of variance (ANOVA), followed by Dunn’s test of pairwise multiple comparisons. We tested correlations between total mass concentrations of CAPs, gaseous pollutants, and endotoxin and glucan concentrations using the nonparametric Spearman rank order correlation. We subtracted preexposure biomarker values from 1-hr and 21-hr postexposure values to adjust for the participant’s day-to-day variations in factors such as daily diet and exposure to ambient pollutants and environmental tobacco smoke that may also contribute to variations in systemic biomarker levels.

We used mixed-effects linear regression models (restricted maximum likelihood estimation) to analyze *a*) the associations between biomarkers and particle concentrations measured in the airstreams; and *b*) the associations between biomarkers and concentrations of endotoxin or glucan collected during exposures. Mixed models accounted for the repeated measures, assuming random participant intercepts and random slopes. We used an autoregressive model of order-one to adjust for serial autocorrelation. The statistical software used was S-PLUS^®^ version 6.2 (TIBCO Software Inc., Palo Alto, CA, USA). Age, sex (binary variable, male = 1), BMI, and season [binary variable, warm season (May–October) = 1] were included in all models. Because temperature was controlled and relative humidity was constant in the testing facility, we did not adjust for them in the models. Endotoxin and glucan were considered part of the particle constituents, and their concentrations were correlated with coarse and fine CAP concentrations (see “Results”). To avoid multicollinearity, we did not include them as covariates in any models with CAPs.

Because gaseous pollutant concentrations varied among exposure scenarios and might affect systemic biomarker levels, for a sensitivity analysis we included gaseous pollutants in the airstream as a covariate in two-pollutant models.

Regression results were expressed as mean change in biomarker concentrations [95% confidence interval (CI)]. A two-tailed value of *p* ≤ 0.05 was considered statistically significant.

## Results

For the study on coarse and fine CAPs, we recruited in total 58 subjects. Eight subjects withdrew from the study, one due to high blood pressure, another due to high blood cholesterol level, and six due to time conflicts. For the ultrafine CAP study, five additional subjects were enrolled to make the total sample size of 25 subjects. In total, 55 female and male participants were enrolled. For the characteristics of the cohort, see Supplemental Material,Table S1.

Table 1 presents air pollutant concentrations in the exposure airstreams of CAP, filtered ambient, and filtered medical air delivered to the participants. The temperature and relative humidity were stable (mean ± SD = 24.0°C ± 1.2°C and 25.0% ± 12.4%, respectively). Gaseous pollutant concentrations in filtered ambient air were not significantly different from those in coarse and fine CAPs. O_3_, NO_2_, and CO concentrations in filtered medical air were significantly lower than in the CAPs exposure air.

**Table 1:** Pollutant concentrations in the exposure airstream.

Exposure/pollutant	*n*	Mean ± SD
Study of coarse and fine CAPs
Filtered ambient air
PM (μg/m^3^)	29	–0.4 ± 13.5
Endotoxin [ln(ng/m^3^)]	29	0.5 ± 0.3
β-Glucan [ln(pg/m^3^)]	29	7.1 ± 1.9
SO_2_ (ppb)	28	1.5 ± 1.7
O_3_ (ppb)	29	12.6 ± 7.3**
NO_2_ (ppb)	26	20.8 ± 12.2**
CO (ppm)	29	0.3 ± 0.1**
Filtered medical air
PM (μg/m^3^)	41	2.0 ± 8.8
Endotoxin [ln(ng/m^3^)]	ND	ND
β-Glucan [ln(pg/m^3^)]	ND	ND
SO_2_ (ppb)	41	1.1 ± 1.0
O_3_ (ppb)	41	1.0 ± 2.1
NO_2_ (ppb)	41	1.2 ± 4.6
CO (ppm)	40	0.5 ± 0.2
Coarse CAP
PM (μg/m^3^)	76	212.9 ± 52.0
Endotoxin [ln(ng/m^3^)]	74	2.0 ± 1.1*
β-Glucan [ln(pg/m^3^)]	61	10.5 ± 2.3*
SO_2_ (ppb)	75	2.5 ± 2.8**
O_3_ (ppb)	76	13.2 ± 7.2**
NO_2_ (ppb)	68	19.5 ± 9.6**
CO (ppm)	75	0.3 ± 0.1**
Fine CAP
PM (μg/m^3^)	29	238.4 ± 62.0
Endotoxin [ln(ng/m^3^)]	28	2.0 ± 0.6*
β-Glucan [ln(pg/m^3^)]	25	9.3 ± 1.1*
SO_2_ (ppb)	28	1.5 ± 1.2
O_3_ (ppb)	29	11.2 ± 6.7**
NO_2_ (ppb)	26	17.4 ± 8.6**
CO (ppm)	28	0.3 ± 0.1**
Study of ultrafine CAP
Filtered medical air
PM (μg/m^3^)	25	8.8 ± 21.1
Particle number (count/cm^3^)	25	16,405 ± 53,152
Endotoxin [ln(ng/m^3^)]	ND	ND
β-Glucan [ln(pg/m^3^)]	ND	ND
SO_2_ (ppb)	25	1.6 ± 1.2
O_3_ (ppb)	25	1.0 ± 1.6
NO_2_ (ppb)	25	3.5 ± 6.8
CO (ppm)	25	0.5 ± 0.2
Ultrafine CAP
PM (μg/m^3^)	25	135.8 ± 67.2**
Particle number (count/cm^3^)	25	227,767 ± 63,902**
Endotoxin [ln(ng/m^3^)]	6	0.12 ± 0.01
β-Glucan [ln(pg/m^3^)]	6	9.0 ± 1.3
SO_2_ (ppb)	25	2.5 ± 1.5**
O_3_ (ppb)	25	5.1 ± 6.2**
NO_2_ (ppb)	25	12.1 ± 8.4**
CO (ppm)	25	0.3 ± 0.2**
ND, not detectable. *Significantly different from filtered ambient air, *p *< 0.05. **Significantly different from filtered medical air, *p *< 0.05.		

Endotoxin and glucan concentrations in coarse and fine CAPs were similar, but significantly higher than in filtered ambient air. The endotoxin and glucan levels in filtered medical air were nondetectable. Endotoxin and glucan concentrations were measured during six ultrafine CAP exposures. Ultrafine CAP appears to contain the lowest concentrations of endotoxin and glucan among the three particle size fractions. Mass concentrations of coarse and fine CAPs were relatively strongly correlated with endotoxin and glucan levels (*r* = 0.75 and 0.76 for endotoxin and glucan in coarse CAP, respectively, *p* < 0.001; *r* = 0.59 and 0.67 for endotoxin and glucan in fine CAP, respectively, *p* < 0.001). Correlations between coarse and fine CAP mass concentrations and gas concentrations measured in the airstream were weak (*r-*values ranging from –0.25 for correlations between coarse CAP and CO to 0.39 for correlations between coarse CAP and NO_2,_ *p* < 0.01; *r-*values for fine CAP verses gases were also within this range).

Table 2 presents the regression results for changes in blood biomarker concentrations per 100-μg/m^3^ increase in CAP mass concentrations. Exposure to coarse CAP was associated with increased VEGF at 1 hr postexposure in two-pollutant models. Adding gases in the models as a covariate strengthened the associations between coarse CAP and VEGF resulting in larger magnitude of effect estimates and smaller *p*-values, suggesting that gases may have moderately confounded the results for coarse CAP; without adjusting for gases in models, the association with coarse CAP might be underestimated. Exposure to coarse CAP was not significantly associated with IL-6, CRP, ET-1, and MDA. Exposure to fine CAP was not consistently associated with any of the blood biomarkers, and ultrafine CAP appears to be negatively associated with VEGF at 21 hr postexposure in single- and two-pollutant models.

**Table 2:** Mean changes in blood biomarker concentrations (95% CI) per 100-μg/m^3^ increase in CAP mass concentration in single- and two-pollutant models.

Model	Coarse CAP		Fine CAP		Ultrafine CAP	
	1-hr postexposure	21-hr postexposure	1-hr postexposure	21-hr postexposure	1-hr postexposure	21-hr postexposure
IL-6 (pg/mL)
CAP alone	0.00 (–0.21, 0.21)	–0.05 (–0.23, 0.12)	–0.05 (–0.14, 0.04)	–0.01 (–0.15, 0.12)	–0.02 (–0.52, 0.48)	–0.05 (–0.32, 0.23)
+ SO_2_	–0.02 (–0.23, 0.20)	–0.10 (–0.28, 0.09)	–0.06 (–0.15, 0.04)	–0.05 (–0.19, 0.10)	–0.02 (–0.53, 0.49)	–0.05 (–0.33, 0.23)
+ O_3_	–0.02 (–0.23, 0.20)	–0.04 (–0.23, 0.15)	–0.05 (–0.15, 0.04)	–0.01 (–0.16, 0.13)	–0.02 (–0.52, 0.49)	–0.01 (–0.27, 0.24)
+ NO_2_	0.02 (–0.21, 0.24)	–0.10 (–0.31, 0.10)	–0.05 (–0.15, 0.05)	–0.04 (–0.19, 0.12)	–0.03 (–0.56, 0.49)	–0.06 (–0.34, 0.23)
+ CO	0.01 (–0.21, 0.22)	–0.04 (–0.24, 0.17)	–0.06 (–0.16, 0.04)	–0.02 (–0.17, 0.13)	0.07 (–0.44, 0.58)	0.00 (–0.28, 0.29)
CRP (ng/mL)
CAP alone	–2.84 (–9.98, 4.31)	–4.12 (–12.73, 4.49)	3.73 (–5.10, 12.56)	–10.98 (–27.71, 5.76)	85.0 (–14.1, 184.2)	–28.2 (–155.3, 98.9)
+ SO_2_	–5.92 (–13.58, 1.74)	–7.15 (–16.39, 2.10)	2.63 (–6.32, 11.58)	–9.40 (–26.69, 7.88)	–62.7 (–460.2, 334.8)	–76.9 (–744.3, 590.5)
+ O_3_	–2.17 (–10.03, 5.69)	–7.78 (–17.51, 1.94)	1.87 (–7.94, 11.68)	–14.07 (–32.37, 4.24)	–63.6 (–442.3, 315.1)	–86.5 (–765.0, 592.0)
+ NO_2_	–0.03 (–8.39, 8.33)	–9.53 (–19.31, 0.26)	3.06 (–6.55, 12.67)	–9.67 (–26.84, 7.50)	–137.4 (–521.6, 246.8)	–43.7 (–742.2, 654.8)
+ CO	–4.37 (–12.00, 3.26)	–3.49 (–12.68, 5.71)	2.38 (–7.06, 11.82)	–15.90 (–35.46, 3.66)	–43.1 (–447.8, 361.6)	–192.5 (–881.8, 496.8)
ET-1 (pg/mL)
CAP alone	–0.01 (–0.06, 0.04)	0.00 (–0.04, 0.05)	0.04 (–0.03, 0.10)	0.00 (–0.04, 0.04)	0.08 (–0.20, 0.37)	–0.02 (–0.21, 0.17)
+ SO_2_	–0.02 (–0.07, 0.03)	0.01 (–0.04, 0.05)	0.03 (–0.04, 0.10)	0.00 (–0.05, 0.04)	0.08 (–0.21, 0.37)	–0.02 (–0.21, 0.18)
+ O_3_	–0.01 (–0.07, 0.04)	0.01 (–0.04, 0.05)	0.04 (–0.03, 0.11)	0.00 (–0.05, 0.05)	0.09 (–0.20, 0.38)	–0.02 (–0.22, 0.17)
+ NO_2_	–0.01 (–0.06, 0.04)	0.01 (–0.04, 0.06)	0.04 (–0.03, 0.11)	0.01 9 (–0.03, 0.06)	0.11 (–0.19, 0.41)	0.00 (–0.21, 0.21)
+ CO	–0.01 (–0.07, 0.04)	0.00 (–0.04, 0.05)	0.05 (–0.02, 0.12)	0.01 (–0.04, 0.05)	0.11 (–0.19, 0.41)	0.00 (–0.21, 0.20)
VEGF (pg/mL)
CAP alone	1.45 (–0.37, 3.28)	–0.01 (–2.77, 2.75)	–0.18 (–2.58, 2.21)	–0.02 (–3.57, 3.53)	–2.25 (–8.10, 3.60)	–8.38 (–17.23, 0.48)*
+ SO_2_	1.63 (–0.27, 3.54)*	–0.23 (–3.08, 2.62)	–0.04 (–2.52, 2.45)	0.72 (–2.98, 4.43)	–2.27 (–8.20, 3.65)	–8.29 (–17.25, 0.68)*
+ O_3_	2.41 (0.41, 4.40)**	1.29 (–1.70, 4.28)	0.50 (–1.98, 2.98)	0.80 (–2.90, 4.50)	–2.31 (–8.22, 3.61)	–8.34 (–17.34, 0.67)*
+ NO_2_	2.14 (0.10, 4.18)**	0.65 (–2.71, 4.01)	–0.09 (–2.71, 2.52)	0.91 (–2.86, 4.68)	–1.69 (–7.97, 4.59)	–6.22 (–15.50, 3.06)
+ CO	1.64 (–0.27, 3.54)*	1.00 (–1.22, 3.23)	0.10 (–2.49, 2.68)	0.54 (–2.34, 3.41)	–1.53 (–7.48, 4.41)	–0.97 (–9.61, 7.67)
MDA (μM)
CAP alone	0.00 (–0.11, 0.12)	–1.93 (–4.84, 0.99)	–0.10 (–0.24, 0.05)	–1.73 (–4.23, 0.76)	0.00 (–0.27, 0.27)	–0.04 (–0.29, 0.20)
+ SO_2_	0.00 (–0.12, 0.12)	–1.91 (–4.83, 1.01)	–0.07 (–0.21, 0.07)	–1.70 (–4.20, 0.81)	0.00 (–0.27, 0.28)	–0.05 (–0.29, 0.20)
+ O_3_	0.00 (–0.13, 0.13)	–1.98 (–4.91, 0.94)	–0.09 (–0.24, 0.06)	–1.73 (–4.25, 0.78)	0.00 (–0.27, 0.27)	–0.06 (–0.29, 0.18)
+ NO_2_	0.00 (–0.13, 0.13)	–1.95 (–4.99, 1.09)	–0.16 (–0.31, –0.01)**	–1.78 (–4.38, 0.81)	–0.02 (–0.31, 0.26)	–0.13 (–0.37, 0.11)
+ CO	0.00 (–0.12, 0.13)	–2.03 (–4.95, 0.89)	–0.07 (–0.22, 0.08)	–1.86 (–4.42, 0.69)	0.03 (–0.25, 0.32)	0.04 (–0.21, 0.28)
*p < 0.1. **p < 0.05.						

**Figure 1:**
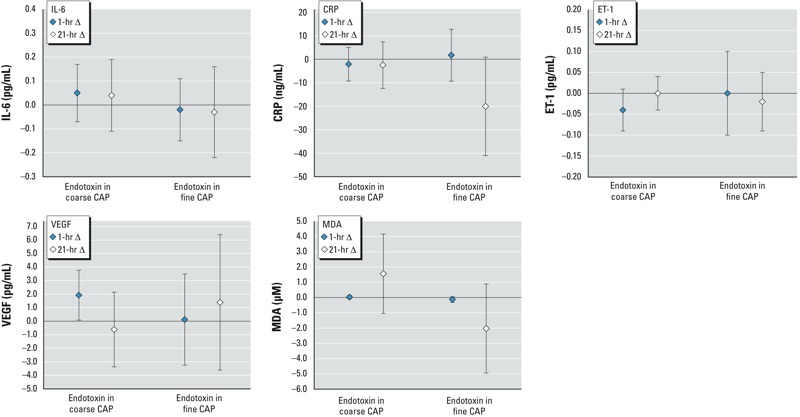
Mean changes in blood biomarker concentrations (95% CI) per unit [ln(ng/m^3^)] increase in endotoxin in coarse or fine CAP. The number of observations was 142–146 and 99 for exposures to endotoxin in coarse and fine CAP, respectively.

Figure 1 presents the associations between endotoxin in CAPs and biomarker concentrations in blood. Endotoxin in coarse CAP was significantly associated with increased VEGF at 1 hr postexposure. Endotoxin was not associated with other blood biomarkers. Glucan was not associated with any of the blood biomarkers (see Supplemental Material, Table S2).

Table 3 shows the regression results for changes in urinary biomarkers per 100-μg/m^3^ increase in CAP mass concentrations. Exposures to coarse and ultrafine CAPs were associated with increased urinary 8-OHdG at 1 hr postexposure, and exposure to fine CAP was associated with increased MDA at 1 hr and 21 hr postexposure, in single- and two-pollutant models. Fine CAP showed a consistent trend of association with urinary VEGF at 1 hr postexposure in single- and two-pollutant models (*p*-values 0.07–0.10). Adding gases in the models as a covariate moderately strengthened the associations between CAPs and urinary biomarkers by rendering smaller *p*-values, but the magnitude of the associations did not change markedly, suggesting that gases may have minor confounding influence over the results for CAPs. Nevertheless, not adjusting for gases might underestimate the strength of the associations. The number count variable for ultrafine CAP yielded similar regression outcomes (see Supplemental Material, Table S3).

**Table 3:** Mean changes in urinary biomarker concentrations (95% CI) per 100-μg/m^3^ increase in CAP mass concentration in single- and two-pollutant models.

Biomarker/model	Coarse CAP		Fine CAP		Ultrafine CAP	
	1 hr postexposure	21 hr postexposure	1 hr postexposure	21 hr postexposure	1 hr postexposure	21 hr postexposure
VEGF (pg/mg creatinine)
CAP alone	8.23 (–2.68, 19.14)	6.61 (–5.34, 18.55)	9.10 (–0.98, 19.17)*	5.60 (–4.78, 15.98)	4.40 (–17.88, 26.69)	9.16 (–12.80, 31.11)
+ SO_2_	7.33 (–3.92, 18.59)	3.52 (–8.95, 16.00)	8.88 (–1.41, 19.18)*	4.39 (–6.52, 15.31)	4.11 (–1.93, 10.15)	8.14 (–12.87, 29.15)
+ O_3_	6.31 (–5.48, 18.11)	6.45 (–6.53, 19.43)	7.47 (–3.09, 18.03)	5.64 (–5.32, 16.60)	4.65 (–2.11, 11.40)	9.39 (–12.70, 31.47)
+ NO_2_	6.87 (–5.66, 19.41)	6.42 (–7.29, 20.13)	9.89 (–1.86, 21.65)	4.31 (–7.22, 15.84)	3.86 (–2.71, 10.43)	16.11 (–6.51, 38.73)
+ CO	10.95 (–0.30, 22.20)*	8.24 (–4.17, 20.65)	10.26 (–0.79, 21.31)*	8.95 (–2.27, 20.16)	2.08 (–4.91, 9.07)	9.12 (–14.04, 32.28)
8-OHdG (ng/mg creatinine)
CAP alone	0.24 (–0.02, 0.50)*	0.01 (–0.26, 0.27)	–0.19 (–0.55, 0.17)	–0.19 (–0.50, 0.13)	0.69 (0.09, 1.29)**	0.19 (–0.41, 0.79)
+ SO_2_	0.29 (0.02, 0.56)**	0.06 (–0.22, 0.35)	–0.20 (–0.58, 0.18)	–0.20 (–0.52, 0.13)	0.72 (0.09, 1.34)**	0.18 (–0.42, 0.78)
+ O_3_	0.22 (–0.07, 0.51)	–0.03 (–0.32, 0.27)	–0.23 (–0.60, 0.15)	–0.18 (–0.51, 0.15)	0.68 (0.09, 1.26)**	0.19 (–0.40, 0.79)
+ NO_2_	0.27 (–0.03, 0.57)*	–0.05 (–0.38, 0.27)	–0.20 (–0.62, 0.21)	–0.33 (–0.67, 0.01)*	0.76 (0.15, 1.38)**	0.22 (–0.40, 0.84)
+ CO	0.30 (0.04, 0.57)**	0.02 (–0.26, 0.30)	–0.12 (–0.50, 0.26)	–0.18 (–0.51, 0.16)	0.57 (0.00, 1.15)**	0.29 (–0.34, 0.91)
MDA (nmol/mg creatinine)
CAP alone	0.07 (–0.09, 0.23)	0.05 (–0.18, 0.29)	0.31 (0.02, 0.60)**	0.27 (–0.04, 0.57)*	0.15 (–0.13, 0.43)	0.29 (–0.20, 0.78)
+ SO_2_	0.09 (–0.08, 0.26)	0.09 (–0.16, 0.34)	0.31 (0.02, 0.60)**	0.23 (–0.07, 0.54)	0.18 (–0.11, 0.48)	0.29 (–0.20, 0.79)
+ O_3_	0.05 (–0.13, 0.23)	0.02 (–0.25, 0.28)	0.36 (0.06, 0.66)**	0.29 (–0.04, 0.62)*	0.15 (–0.13, 0.44)	0.29 (–0.20, 0.78)
+ NO_2_	0.01 (–0.18, 0.19)	–0.03 (–0.29, 0.24)	0.24 (–0.08, 0.57)	0.21 (–0.13, 0.55)	0.11 (–0.19, 0.41)	0.28 (–0.25, 0.80)
+ CO	0.06 (–0.11, 0.23)	0.04 (–0.21, 0.29)	0.31 (0.00, 0.63)**	0.26 (–0.08, 0.59)	0.10 (–0.22, 0.41)	0.26 (–0.26, 0.78)
*p < 0.1. **p < 0.05.						

**Figure 2:**
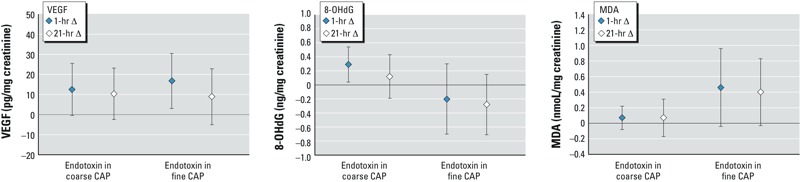
Mean changes in urinary biomarker concentrations (95% CI) per unit [ln(ng/m^3^)] increase in endotoxin in coarse or fine CAP. The number of observations was 143 and 98 for exposures to endotoxin in coarse and fine CAP, respectively.

Figure 2 shows the associations between endotoxin in CAPs and urinary biomarkers. Endotoxin in coarse and fine CAPs was associated with increased urinary VEGF at 1 hr postexposure. Endotoxin in coarse CAP was associated with increased 8-OHdG at 1 hr postexposure. Exposure to endotoxin in fine CAP was associated with increased urinary MDA at 1 hr and 21 hr postexposure at *p*-values 0.08 and 0.07, respectively. Glucan was not associated with any urinary biomarkers (see Supplemental Material, Table S2).

## Discussion

Our results demonstrate that exposure to coarse CAP was associated with increased blood VEGF and urinary 8-OHdG concentrations, whereas exposure to fine CAP was associated with increased urinary VEGF and MDA concentrations. Exposure to ultrafine CAP was associated with increased urinary 8-OHdG. These biomarker changes were transitory, lasting < 21 hr postexposure. These associations were robust after adjusting for age, sex, BMI, and season in the models. Adding gaseous pollutants in the models typically strengthened the associations by reducing *p*-values and slightly increasing the magnitude of effect estimates, suggesting that these gases may have moderately confounded the results for CAPs. The confounding possibly was attributable to the gases not removed in the airstreams of filtered ambient air and CAPs. Not adjusting for these gases in the models might underestimate the effects of CAPs. All results taken together, on an equal mass concentration basis (per 100 μg/m^3^), the three particle size fractions affected blood and urinary biomarkers in different ways: Coarse CAP appears to have more influence on blood VEGF, fine CAP more influence over urinary MDA, and ultrafine CAP more effect on urinary 8-OHdG. Regression results for endotoxin mirrored those found in coarse or fine CAPs where endotoxin was collected, suggesting that endotoxin in CAPs contributed to systemic changes in vascular mediators and oxidative stress.

We used VEGF and ET-1 as biomarkers of vascular function, and found that coarse CAP and the endotoxin constituent in coarse and fine CAPs were associated with a transient increase in blood and urinary VEGF levels. VEGF regulates the mobilization of endothelial progenitor cells from bone marrow to an injured site (Haberzettl et al. 2012). Increased VEGF levels may represent an acute systemic response to endothelial injury during exposure to PM and endotoxin. Daily exposure to indoor and outdoor PM_2.5_ and black carbon has been associated with increased blood VEGF levels in seniors (Liu et al. 2009b). To our knowledge, the present study is the first to report an association between exposure to ambient PM and endotoxin and elevated urinary VEGF levels. ET-1 is an endogenous vasoconstrictor (Haynes et al. 1996). Elevated plasma ET-1 was found in children who were chronically exposed to air pollution in Mexico City (Calderón-Garcidueñas et al. 2007) and in seniors on high-pollution days (Liu et al. 2009b). In the present study, we did not observe changes in blood ET-1. This discrepancy might be attributable to differences in the participants’ demographics and health status compared to other cited studies.

Overproduction of IL-6 induces immune-mediated inflammatory diseases (Nishimoto and Kishimoto 2006). CRP is an acute-phase reactant marker for underlying systemic inflammation and a strong predictor for atherosclerosis, coronary artery disease, and myocardial infarction (Anderson et al. 1998). In the present study, CAPs were not associated with any changes in blood IL-6 and CRP. There is conflicting evidence in the literature about the influence of air pollution on blood IL-6 and CRP. Increased IL-6 and/or CRP were reported in a controlled exposure study (Urch et al. 2010) and epidemiological studies (Delfino et al. 2009). Other studies found either no association between exposure to PM and IL-6 and CRP (Behbod et al. 2013; Bräuner et al. 2008; Liu et al. 2009b), or an association between PM and a reduction in CRP levels (Rückerl et al. 2007). These discrepancies among studies may be attributed to different characteristics of study participants or different particle compositions. It is also possible that CRP and IL-6 were not very sensitive to a 130-min exposure to PM.

8-OHdG is a by-product from damaged DNA when it is attacked by hydroxyl free radicals at deoxyguanosine in DNA (Park and Floyd 1992). We found significant associations between urinary 8-OHdG at 1 hr postexposure and coarse and ultrafine CAPs, and endotoxin collected with coarse CAP. These findings are consistent with a previous report that elevated ambient ultrafine particle concentrations were associated with increases in children’s urinary 8-OHdG (Song et al. 2013). MDA is formed during oxidative degradation of cellular lipids (Janero 1990). Our results show that urinary MDA was associated with exposure to fine CAP and endotoxin collected with fine CAP. Fine particulate pollutants have been demonstrated to cause formation of excessive amount of reactive oxygen species in airways and the cardiovascular system in experimental animals, leading to tissue inflammation and cell death (Dye et al. 1997). Our results provide further supporting evidence that ambient coarse, fine, and ultrafine PM may facilitate oxidative damage to DNA or membrane lipids.

We found that endotoxin from CAPs was associated with increases in blood and urinary VEGF, and urinary MDA and 8-OHdG. In an early study, we reported that endotoxin was associated with increased blood leucocytes and neutrophils 24 hr postexposure, regardless of the origin (coarse or fine CAP) of endotoxin (Behbod et al. 2013). These findings suggest that endotoxin as a component of outdoor PM may contribute to damage to important macromolecules leading to tissue injury. Although the levels of endotoxin in coarse and fine CAPs were similar, exposures to coarse and fine CAPs themselves were not associated with the same changes in biomarkers. Moreover, endotoxin in coarse and fine CAPs was associated with different biomarker responses. One explanation for this may be that the differences in airway deposition location and rates for coarse and fine CAPs (as well as endotoxin carried by them) may have influenced their effects on biomarkers. It is also possible that other constituents in CAPs such as metals and organic carbon may have contributed to the effects by CAPs as well. We have archived CAP samples for future investigations of PM constituents.

In conclusion, in this study we found that a 130-min exposure to concentrated ambient PM was associated with changes in blood and urinary biomarkers for vascular function and oxidative stress that influenced DNA and cellular lipid integrity in humans. The three size fractions of CAPs appear to affect these biomarkers in a different manner, with coarse CAP having a stronger association with VEGF in blood, fine CAP having stronger association with urinary marker of lipid peroxidation, and ultrafine CAP having a stronger association with urinary marker of DNA oxidative damage. Endotoxin constituents for coarse and fine CAPs were significantly associated with systemic changes in VEGF and the biomarkers of DNA and lipid oxidation, suggesting that endotoxin played a role in the effects of coarse and fine CAPs on human health.

## Supplemental Material

(275 KB) PDF
