# Supplementary material for: Effects of Ambient Coarse, Fine, and Ultrafine Particles and Their Biological Constituents on Systemic Biomarkers: A Controlled Human Exposure Study
Source: Environ Health Perspect. 2015 Jan 16;123(6):534–40. doi: 10.1289/ehp.1408387 (PMC4455587; doi:10.1289/ehp.1408387)
Supplement: (275 KB) PDF [file ehp.1408387.s001.508.pdf]

Supplemental Material

Effects of Ambient Coarse, Fine, and Ultrafine Particles and Their Biological Constituents on Systemic Biomarkers: A Controlled Human Exposure Study

Ling Liu, Bruce Urch, Raymond Poon, Mieczyslaw Szyszkowicz, Mary Speck, Diane R. Gold, Amanda J. Wheeler, James A. Scott, Jeffrey R. Brook, Peter S. Thorne, and Frances S. Silverman

Table S1. Baseline characteristics of subjects (N=55).

Characteristic	Median (5th – 95th percentile, unless otherwise noted)
Age, years (mean ± standard deviation)	28 ± 9
Sex (female/male)	29/26
Race (Asian/Caucasian/other)	24/23/8
Body mass index (kg/m^2)	23.0 (19.5, 27.8)
Blood biomarkers	
IL-6 (pg/ml)	0.8 (0.2, 3.3)
CRP (µg/ml)	0.43 (0.02, 5.38)
ET-1 (pg/ml)	1.2 (0.6, 2.4)
VEGF (pg/ml)	27.5 (11.2, 65.1)
MDA (µM)	0.8 (0.3, 2.9)
Urinary biomarkers	
VEGF (pg/mg Creatinine)	54.4 (1.2, 176.1)
8-OHdG (ng/mg Creatinine)	3.7 (1.6, 10.6)
MDA (nmol/mg Creatinine)	2.2 (1.1, 5.4)

Table S2. Mean changes in biomarker concentrations (95% confidence interval) in blood and urine for an increase in one unit [ln(pg/m^3)] of glucan in coarse or fine CAP.

**Biomarker**	**Glucan in coarse CAP 1-hr post exposure**	**Glucan in coarse CAP 21-hr post exposure**	**Glucan in fine CAP 1-hr post exposure**	**Glucan in fine CAP 21-hr post exposure**
**Blood biomarkers**				
IL-6 (pg/ml)	0.00 (-0.14, 0.15)	-0.04 (-0.10, 0.03)	-0.05 (-0.11, 0.00)*	Not available[a]
CRP (ng/ml)	-1.01 (-4.23, 2.20)	-1.66 (-5.67, 2.35)	-0.38 (-4.86, 4.09)	-4.38 (-14.87, 6.10)
ET-1 (pg/ml)	-0.01 (-0.03, 0.01)	0.00 (-0.02, 0.02)	0.03 (-0.02, 0.08)	0.01 (-0.02, 0.05)
VEGF (pg/ml)	0.72 (-0.47, 1.91)	-0.30 (-1.74, 1.14)	-0.89 (-2.64, 0.86)	-0.83 (-3.48, 1.81)
MDA (µM)	-0.01 (-0.06, 0.04)	0.82 (-0.43, 2.07)	-0.09 (-0.19, 0.02)	-1.19 (-2.74, 0.37)
**Urinary biomarkers**				
VEGF (pg/mg Creatinine)	4.05 (-2.36, 10.46)	0.77 (-4.23, 5.77)	2.79 (-5.10, 10.68)	4.64 (-3.86, 13.14)
8-OHdG (ng/mg Creatinine)	0.09 (-0.04, 0.21)	0.02 (-0.13, 0.17)	-0.06 (-0.34, 0.22)	0.00 (-0.24, 0.24)
MDA (nmol/mg Creatinine)	0.02 (-0.05, 0.09)	0.04 (-0.07, 0.15)	0.12 (-0.12, 0.35)	0.09 (-0.16, 0.33)

[*]$p<0.1$. [a]Result not available due to singularity problem of the matrix in the model.

Table S3. Mean changes in biomarker concentrations (95% confidence interval) in blood and urine for exposure to ultrafine CAP. Ultrafine CAP was in number count concentration ($100,000/cm^3$).

Biomarker	1-hr post exposure	21-hr post exposure
**Blood biomarkers**		
IL-6 (pg/ml)	0.10 (-0.29, 0.49)	0.08 (-0.12, 0.28)
CRP (ng/ml)	26.2 (-27.1, 79.4)	-10.0 (-88.4, 68.4)
ET-1 (pg/ml)	0.00 (-0.20, 0.20)	0.02 (-0.08, 0.12)
VEGF (pg/ml)	-1.00 (-4.92, 2.92)	-2.90 (-8.78, 2.98)
MDA (µM)	2.00 (-3.88, 7.88)	-0.02 (-0.18, 0.14)
**Urinary biomarkers**		
VEGF (pg/mg Creatinine)	7.00 (-8.68, 22.68)	0.22 (-13.90, 14.33)
8-OHdG (ng/mg Creatinine)	0.30 (-0.09, 0.69)*	0.20 (-0.19, 0.59)
MDA (nmol/mg Creatinine)	0.10 (-0.10, 0.30)	0.27 (-0.12, 0.66)*

*$p<0.1$.
